# Bond strength and elemental analysis of oxidized dentin bonded to resin modified glass ionomer based restorative material

**DOI:** 10.4317/jced.55432

**Published:** 2019-03-01

**Authors:** Youssef-Abdullah Al-Gerny, Sayed-Mohammed Ghorab, Tarek-Ahmed Soliman

**Affiliations:** 1 Restorative Dental Science Department, Faculty of Dentistry, King Khalid University, Abha, KSA; 2 Dental Biomaterials Department, Faculty of Dentistry, Mansoura University, Mansoura, Egypt

## Abstract

**Background:**

This study aimed to investigate the influence of hesperidin application on the bonding of resin-modified glass ionomer-based restorative material to dentin treated with oxygen-induced endodontic irrigants.

**Material and Methods:**

One hundred human permanent molars were categorized into five groups (n= 20/group), treated with various irrigants as follows: Group C; distilled water (control group), Group SH; 5.25% NaOCl, Group SH+H; 5.25% NaOCl + 5 % hesperidin, Group HP; 10% H2O2, Group HP+H; 10% H2O2 + 5 % hesperidin. Specimens were bonded with RMGI based restorative material. For each group, half of the specimens were evaluated for µSBS by a universal testing machine and the other half for dentin ion uptake by EDX. Additional ten specimens (n=2/per group) were prepared for the micro-morphological analysis under SEM.

**Results:**

Hesperidin groups improved the µSBS, with a significant effect for HP+H group (*p*<. 05). Dentin ion uptake was significantly (*p*<. 05) improved in hesperidin groups.

**Conclusions:**

In conclusion, Application of hesperidin in conjunction with RMGI based restorative material improved the dentin bond strength and ion uptake; this could be a promising approach to aid dental practitioners in their decisions, regarding which restorative material to use especially in caries susceptible patients.

** Key words:**Hesperidin, elemental analysis, bond strength.

## Introduction

Immediate restoration of endodontically treated teeth is clinically important to prevent bacterial leakage from oral cavity, to withstand occlusal force, and to avoid fracture of remaining tooth structure (1). Core buildup restorative materials have been widely used especially when using the prefabricated posts in the endodontically treated teeth. These materials include three main groups: silver amalgam, resin composite, and resin modified glass ionomer (RMGI) (2). RMGI-based restorative material considers many advantages such as; thermal expansion similar to tooth structure, decreased microleakage, chemical bonding to tooth structure and fluoride release. RMGI primer is a light-cured liquid, designed for resin-modified glass ionomer-based restorative material and applied before placement of the restoration to modify the smear layer and adequately wet the tooth surfaces to facilitate the adhesion (2,3).

Sodium hypochlorite (NaOCl) and hydrogen peroxide (H2O2) are well known as endodontic irrigants due to their antibacterial and de-proteinization effects (4-6). Nevertheless, these irrigants could change the dentin composition and affect its interaction with the restorative materials. Previous studies (4-8) stated that dentin bond strength could be negatively affected by NaOCl application. However, other studies showed neutral (9-11) or even positive results (12-15) depending on the application time and the adhesive type. If the bond strength was decreased because of the oxidizing effects of these irrigants, utilizing a biocompatible antioxidant before resin bonding may reverse this reaction (5).

Hesperidin (HPN), a flavonoid extracted from citrus fruits. HPN yields a wide range of benefits such as: anti-inflammatory, anti-microbial, collagen cross-linker, resistance to caries progression, promotion of the remineralization process and anti-oxidant effects (16,17). There is a lack of information regarding the effect of hespiridin on the interaction between RMGI- based restorative material and the dentin treated with oxygen-induced endodontic irrigants. Accordingly, the primary rationale of this study was to assess the impact of hesperidin application on the bonding of RMGI-based restorative material to dentin treated with oxygen-induced endodontic irrigants. In addition, to determine if the application of HPN has an effect on the chemical structure change of dentin along side RMGI based restorative material. The null hypothesis for this study was that; the use of HPN has no effect (1) on the bond strength and (2) on the chemical structure of NaOCl or H2O2 treated dentin bonded to RMGI based restorative material.

## Material and Methods

RMGI-based restorative material and a 5 % hesperidin solution (prepared by adding 

5 gm HPN powder to its dissolving solution) were used in this study.The chemical composition of the materials used in this study is presented in  Table 1.

**Table 1:**
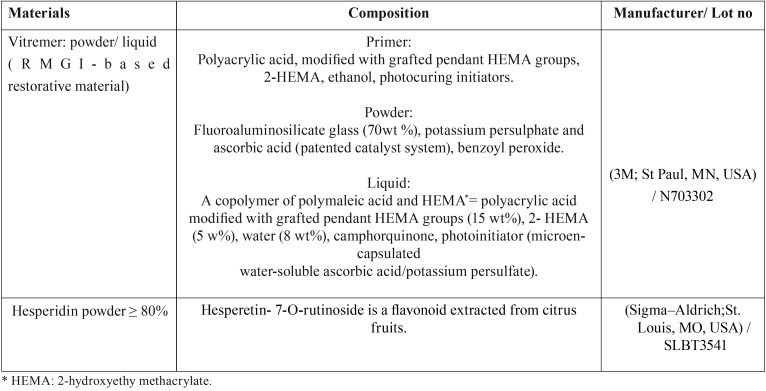
Materials used in the study.

-Study Design

One hundred sound, non-carious human permanent molars were used for µSBS testing (n=50) and for elemental analysis (n=50). This study was approved by the ethical committee for human experiment at Faculty of Dentistry, Mansoura University. For each test, the teeth were subdivided into five groups (n=10/group) according to the type of irrigants used. Additional ten specimens (n=2/group) were used for the micro-morphological analysis of the bonded interface.

-Preparation and Grouping of Specimens

Fifty sound, extracted human non-carious permanent molars were collected and stored in 0.5% chloramine T solution at 4º C for less than 4 wks. The crowns were sectioned 1 mm apical to CEJ. The occlusal one-third were grounded to expose a flat dentin surface using a water-cooled low speed saw (ISOMET, Techcut4, Allied, USA). The flat dentin surfaces were wet polished using 600-grit silicon carbide paper (BuehlerMet II 600, Buehler) to attain a standardized smear layer. The dentin specimens were then embedded in acrylic resin blocks (Vertex; Vertex-dental, Ziest, Netherland) leaving their occlusal surfaces exposed to various types of irrigants and bonding. Specimens were randomly assigned into five groups (n=10) according to the various types of irrigants used as follows: group C: irrigated with distilled water for 30 sec (negative control); group SH: irrigated with 5.25% NaOCl (positive control) (Chlora Extra; CERKAMED, Kwiatkowskiego, Stalowa Wola, Polska) for 30 sec and then rinsed with distilled water for 30 sec; group SH+H: irrigated with 5.25% NaOCl for 30 sec, rinsed with distilled water for 30 sec, and then rinsed with 5 % HPN solution for 10 min; group HP: irrigated with 10% H2O2 (Dimamedical, Cairo, Egypt) for 30 sec and then rinsed with distilled water for 30 sec; group HP+H: irrigated with 10% H2O2 for 30 sec, rinsed with distilled water for 30 sec, and then rinsed with 5% HPN solution for 10 min. The 5% HPN solution was prepared by adding 5 gm of HPN powder in 100 ml of dimethyle sulfoxide (Fisons Equipment; Bishop Meadow Road, Loughborough, UK) under supervision of the Faculty of Pharmacy, Mansoura University.

-Bonding Procedures

Following the treatment procedures, RMGI-based restorative material was applied according to the manufacturer’s instructions: the primer was applied for 30 sec, followed by 15 sec of air-drying. Cylinders were cut from tygon tubes 0.8 mm diameter and 0.5 mm height (Norton Performance Plastic; Cleveland, OH, USA) and mounted on the dentin surface to restrict the bonding area (6). The primer was cured for 20 sec using Elipar FreeLight 2 (3M; St Paul, MN, USA, light output: 1226 mW/cm2). The RMGI-based restorative material was manipulated in 2.5:1 P/L ratio, packed into the tygon tubes using disposable tips and a syringe and light cured for 40 s. A surgical blade was used to remove the tygon tube. The specimens were then coated with a protective varnish (Ketac glaze; 3M; St Paul, MN, USA) prior to storage in distilled water at 37o C for 24 hrs.

-Microshear Bond Strength (µSBS) Test

The microshear bond strength was measured using a universal testing machine (Lloyd Instruments; Fareham, UK). A wire loop with 0.2 mm diameter was inserted and gently flushed around RMGI/dentin interface through half of its circumference. The RMGI/dentin interface and the wire loop were aligned as straight as possible to the load cell of the testing machine. The shear force was applied at a crosshead speed of 0.5 mm/min until failure occurred (18). The de-bonded specimens were inspected under an optical stereomicroscope (Olympus SZ61, Tokyo, Japan) at 20 x magnification to determine the mode of failure, either cohesive in RMGI, mixed or adhesive failure.

-Micro-morphological Examination of theBonded Interface

Ten additional specimens (n=2/group) were prepared, treated as previously mentioned in µSBS testing and restored with the RMGI-based restorative material in a 2 mm layer thickness. The specimens were then sectioned perpendicular to the bonded interface. Successive grits (600, 800, and 1200-grit) of wet silicon carbide papers (MicrocutTM, Buehler, Lake Bluff, USA) were used to polish the cut surfaces. The polished surfaces were then immersed in 10% phosphoric acid, followed by 5% NaOCl for 5 min and cleaned ultrasonically in distilled water (19). The specimens were air-dried, gold sputtered-coated and inspected under the SEM (JSM-6510LV, JOEL, Tokyo, Japan) with 15 KV accelerating voltages (ACCV) at original magnification 2000 x. The RMGI primer /dentin interface was evaluated regarding the presence or absence of smear layer, hybrid layer formation and resin tubular penetration.

-Elemental Analysis

An end cutting diamond bur was used to prepare occlusal cavities (2 mm in diameter and 1 mm depth in dentin) in fifty sound, extracted non-carious permanent molar. A nail varnish was used to coat all surfaces except the cavity preparations. The specimens were then assigned into five groups, treated in the same manner as for µSBS testing and restored with the RMGI-based restorative material. After that, the specimens were stored in a separate 5 ml vial containing distilled water at 37° C for two wks (20). The specimens were then sectioned through the center of the restoration in a bucco-lingual direction using a low-speed diamond disc under water-cooling. Successive grits of wet silicon carbide papers (600, 800, and 1200-grit) were used to polish the sectioned halves. The polishing was then completed using a lapping diamond paste (Diamat, Pace Technologies, Tuscon, AZ, USA). The specimens were placed on stainless steel stubs and examined by the energy dispersive X-ray (EDX). The weight percentages of fluoride, strontium, silicon, and aluminum ions were calculated axially in close vicinity to the adhesive-restoration/tooth interface (21). Three readings of ions weight percentages were averaged to get one from each specimen.

-Statistical Analysis

The µSBS values and ion weight percentages were first checked by the Shapiro–Wilk test for the normal distribution of data and then analyzed using One-way ANOVA and Tukey’s multiple comparison test (*P* < .05). The fracture pattern was analyzed by the Chi-square (χ2) test. Additionally, µSBS data were entered into a Weibull analysis to calculate Weibull modulus, characteristic bond strength, correlation coefficient and the µSBS at 95, 90 and 5% survival probability. The Weibull distribution is given by Ps = EXP [-(σ/σ0)m] where Ps is the survival probability at any shear stress, σ is the µSBS at a given Ps, σ0 is the characteristic microshear bond strength and m is the shape parameter (Weibull Modulus). Ps is obtained by the relation: Ps = k / (N+1) where k is the rank order and N is the group specimen numbers.

## Results

-Microshear Bond Strength (µSBS)

The mean and standard deviations of the µSBS values (MPa) are presented in  Table 2.

**Table 2:**
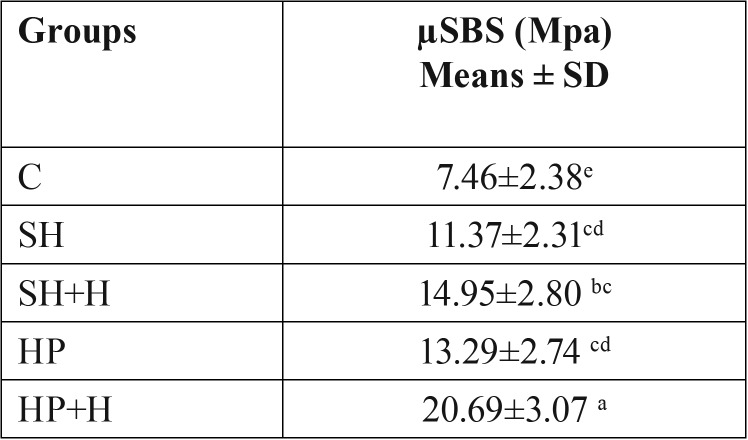
Means ± standard deviations of the microshear bond strength for the different groups in MPa.

**Figure 1:**
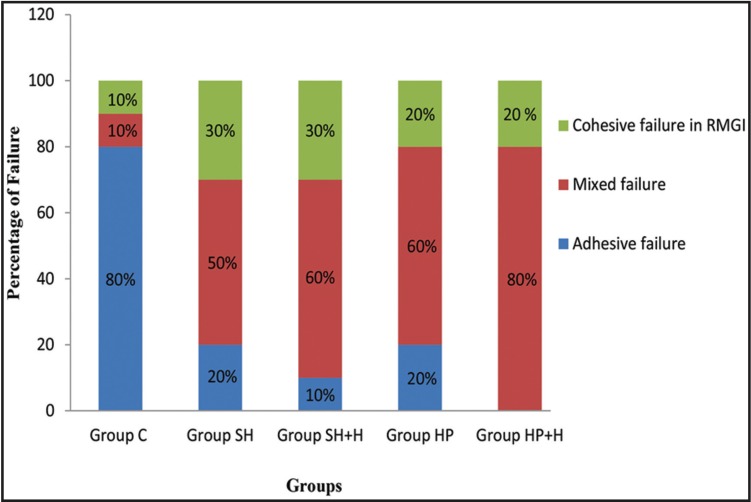
Percentage distribution of failure modes for the different groups.

**Figure 2:**
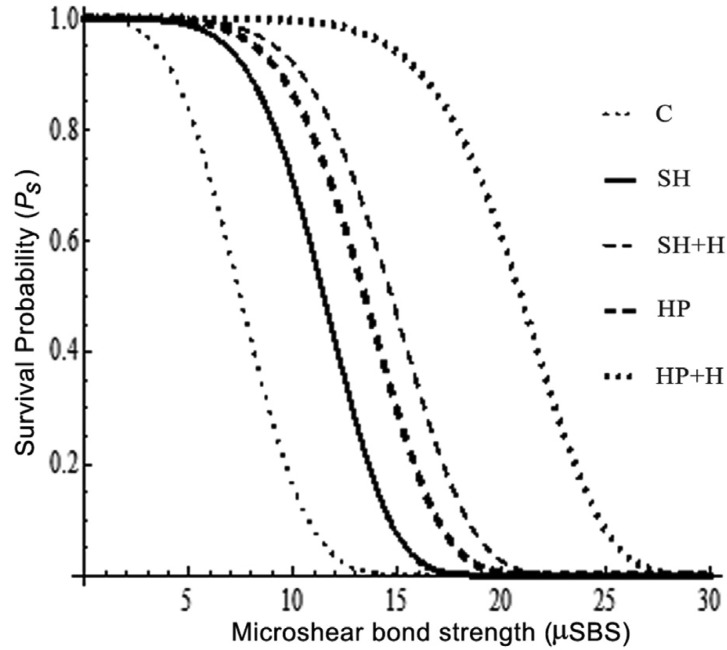
Weibull survival probability (Ps) curve for the different groups.

A significant difference was recorded between the different groups (*P* = .000). The Tukey’s multiple comparison test showed that, the µSBS was improved after hesperidin application with only significant effect in HP+H group (SH+H group, *P*= 0.076 and HP+H group, *P*= .000). There was no significant difference between SH and HP groups (*P*= .49) or in between SH and SH+H (*P*= .07). Figure 1 showed the fracture analysis for each group. Mixed failure was the predominant type (χ2= 10.7, *P*= .03) in all groups except group C, whereas the adhesive failure was the most significant one in this group (χ2= 20.37, *P*= .004).  Table 3 shows the Weibull parameters for all the groups. The Weibull modulus values varied with different treatments, showing higher value for HP+H group. In addition, the survival probability plots for the different groups are presented in figure 2.

**Table 3:**
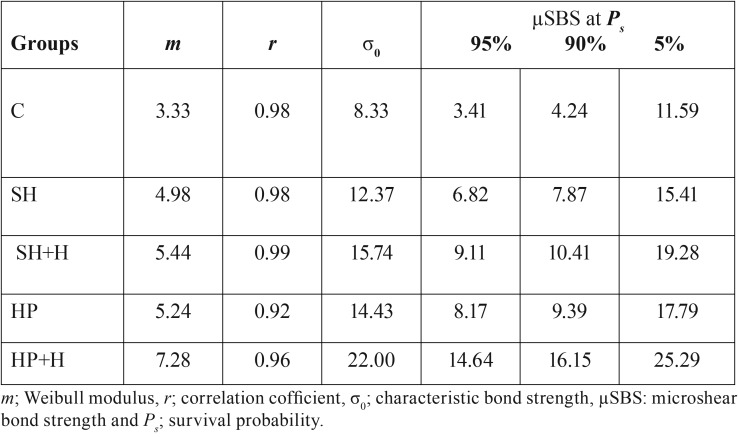
Weibull parameters for the different groups.

-Micro-morphological Analysis of the Bonded Interface

**Figure 3:**
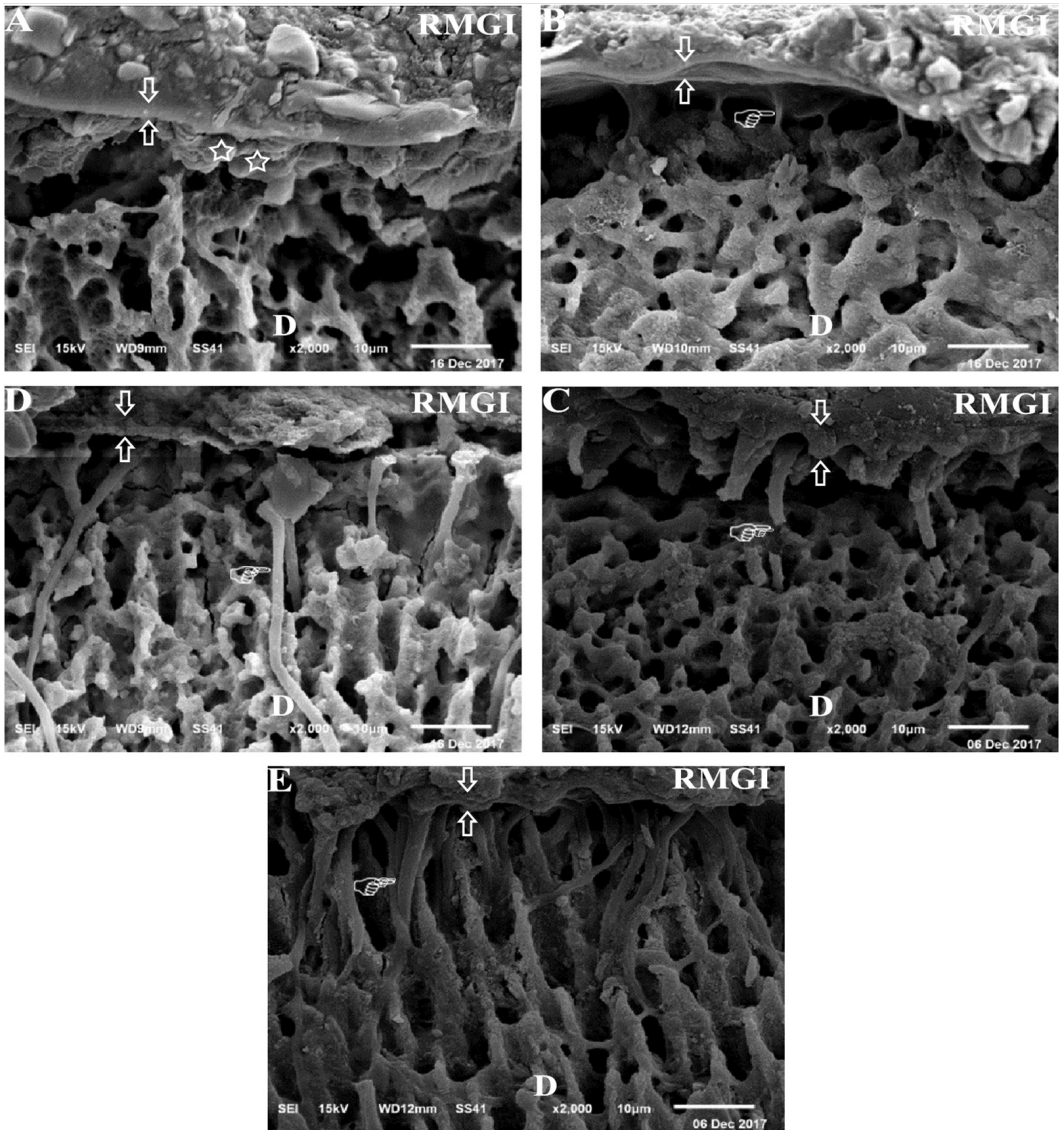
SEM micrographs of RMGI/ dentin interface for all the tested groups after acide/base challenge. Formation of the thick hybrid layer (between arrows) could be observed in all groups (A,B,C,D and E). The deepest part of the smear layer (star) could be seen only in C group (A). Regarding the RMGI primer tubular penetration, there was no penetration in C group (A), limited penetration in SH group (B), more penetration in the HP group (D). After application of hesperidin, the tubular penetration was enhanced in SH+H group and HP+H groups. Nevertheless, there is a disruption along its length in SH+H group. A complete penetration without disruption was observed in HP+H group. Hand pointer showed the different configurations of RMGI primer tubular penetration. RMGI: resin modified glass ionomer based restorative material; D: dentin.

SEM images of dentin/RMGI primer interface are presented in Figure 3. The microscopic evaluations showed wide variations between the groups. Hesperidin groups showed more tubular penetration by RMGI primer.

-Elemental Analysis

The ion weight percentages (mean and standard deviations) of fluoride, strontium, silicon, and aluminum are presented in  Table 4. Significant differences (*P*< .001) were detected for each element among the tested groups. A significant increase in ion weight percentages was detected in hesperidin groups (SH+H and HP+H) in comparison to C, SH and HP groups (*P*< . 001).

**Table 4:**
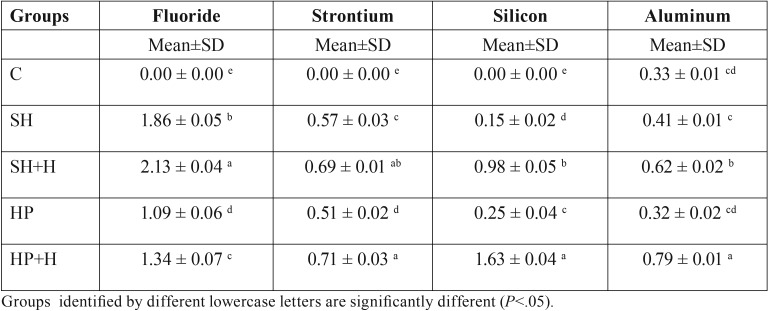
Mean and SD of ion Wt % for fluoride, strontium, silicon, and aluminum in dentin.

## Discussion

In this study, NaOCl and H2O2 were used as irrigants for 30 s as it has been reported that; 30 s irrigation by 5.25% NaOCl eliminate Enterococcus faecalis, which is the most resistant microorganism in root canal (22). HPN was dissolved in dimethyl sulfoxide solutionas it has a low solubility in water (23). A 5% HPN solution was used in this study, as a 5% HPN-containing primer had been reported to improve the immediate bond strength and preserve the bonding durability (24,25).The microshear testing was used in this study. It controls the bonded area and eliminates the pre-stressing factors, such as specimens sectioning, which is performed in the microtensile bond strength test (26,27). In the current study, superficial, sound, and flat dentin surfaces were used as dentin substrate, as it has been reported in other studies (26,27).

The effect of NaOCl and H2O2 on the dentin bond strength showed conflict outcomes as either increased (12-15), decreased (4-8) or even neutral (9-11) results. In the current study, SH and HP groups showed significantly higher µSBS than group C. This could be attributed to the tubular penetration by RMGI primer and formation of resin tags in SH and HP groups (Fig. 3B,D) respectively. On the other hand, no tubular penetration was observed in group C (Fig. 1A). These results are in accordance with the previous studies (12-15), which stated that; NaOCl and H2O2 deproteinize the smear layer-covered dentin by dissolving its organic phase giving rise to an appropriate substrate for bonding. However, other

 studies (9,12,13) reported that, application of NaOCl or H2O2 for 10 minutes could negatively affect the dentin bond strength due to their oxidative property to dentin collagen matrix. Therefore, the effect of oxygen induced endodontic irrigants depends on the application time.

The first null hypothesis that application of hesperidin has no effect on the bond strength of NaOCl or H2O2 treated dentin bonded to RMGI based restorative material was rejected since, HPN improved the µSBS,with significant effect in HP+H group. HPN enhances the cross linking of type I collagen in dentin, which is an essential step in improving the adhesion (16,17,24,25). The pyridinoline crosslinks in type I collagen could be disrupted by NaOCl not by H2O2 (28). Accordingly, HPN could not significantly improve the bond strength of SH+H group. This clarification might be supported through the widespread tubular penetration by RMGI primer in HP+H group (Fig. 3E). However, the tubular penetration was disrupted in SH+H group (Fig. 3C). The fracture analysis showed that, mixed failure was the predominant type in all groups except in group C. The high percentages of mixed failure, especially in HP+H group could be attributed to the better bonding between the primer and dentin substrate and the weak cohesive strength of RMGI material (19).

A weak correlation could exist between the successful laboratory bonding results and the clinical success since several factors could affect the obtained results such as; specimen preparation, materials handling and specimen storage. Accordingly, Weibull analysis proved to be a better predictor of clinical performance of laboratory bonding results before conducting the clinical studies (29). It has been reported that material with higher Weibull modulus is known to have a high 24-hours bond strength (30). HP+H group recorded the highest Weibull modulus (7.28). Furthermore, it showed satisfactory µSBS value (14.64) at 95% survival probability.

Elemental analysis was assessed by energy dispersive x-ray (EDX) since it is both 

a sensitive and an accurate detecting method that is used to analyze the chemical composition and distribution of various elements (31). The weight percentages of fluoride, strontium, aluminum, and silicon ions were investigated in this study since it has been proven that these ions released and exchanged at the tooth interface (20,21). Moreover, these ions are able to enhance the remineralization adjacent to the tooth/restoration interface (21). Hesperidin groups showed an increase in the weight percentages of these ions, which could enhance the remineralization process. The results of the current study are in agreement with a previous study (32) which stated that HPN might have the potential to promote the remineralization process through increasing dentin ion uptake. Accordingly, the second null hypothesis was rejected.

One of the limitation of this study is that it does not undergo aging to test the durability of the bond strength data. In addition, the clinical performance assessment is required to provide reliable recommendations for this in vitro study. Furthermore, ten specimens for Weibull analysis were not sufficient to obtain a reliable conclusion. However, it is thought that the Weibull analysis results have some valuable validity.

Within the limitation of this study, by combining the outcomes from the µSBS, the micro-morphological and elemental analysis, application of hesperidin improved the dentin bond strength and increased the ion weight percentages in dentin along side RMGI based restorative material; this could be a promising approach to aid dental practitioners in their decisions, regarding which restorative material to use especially in caries susceptible patients.
